# Consumer Reactions to *E. Coli* and Antibiotic Residue Recalls: Utility Maximization vs. Regret Minimization

**DOI:** 10.3389/fvets.2020.00611

**Published:** 2020-09-03

**Authors:** Elliott J. Dennis, Kayode Ajewole, Jason S. Bergtold, Ted C. Schroeder

**Affiliations:** ^1^Department of Agricultural Economics, University of Nebraska-Lincoln, Lincoln, NE, United States; ^2^National Institute of Antimicrobial Resistance Research and Education, Ames, IA, United States; ^3^United States Department of Agriculture Economic Research Service, Washington, DC, United States; ^4^Department of Agricultural Economics, Kansas State University, Manhattan, KS, United States; ^5^Center for Risk Management Education and Research, Kansas State University, Manhattan, KS, United States

**Keywords:** antibiotic residue, *E. coli*, food recall, discrete choice analysis, random regret minimization, random utility maximization

## Abstract

Food safety remains a major issue to many consumers. Previous studies examining the economic impact of food safety recalls have focused on Class I recalls. Antibiotic residue in meat products, a Class II recall, has increased in consumer importance yet little is known about how much research and development expenditure should be allocated to reduce antibiotic residue pre- and post-harvest. This study compares demand elasticities and the decrease in willingness to pay in response to either an *E. coli* (Class I) or antibiotic residue (Class II) recall. We compare and contrast two competing behavioral frameworks, Random Utility and Regret Minimizing. Modeling behavior using the random regret framework is found to be more powerful for assessing consumer responses. In addition, we explore if different groups of consumers exist that either maximize utility or minimize regret. Consumer devaluations of *E. coli* (Class I) are 40–65% larger than antibiotic residue (Class II). Approximately 60% of consumers are identified as regret minimizers and 40% were identified as utility maximizers. While industry response and government policy recommendations differed conditional on modeling framework, the regret minimizing framework required smaller price discounts than regret minimizing to maintain the same level of market share.

## Introduction

Consumers trust government regulatory agencies to ensure food products are safe to eat and to publicize product recall notifications when food safety breaches occur. Potential long-term economic losses along the food supply chain due to food safety events are particularly concerning given potential loss of consumer confidence. Furthermore, recalls can endure for several months, as product hazards can take time to identify and trace. By the time that these tasks have been completed, many products already have been shipped and sold to consumers and may never be recovered via a food recall^1^. Several studies have estimated economic impacts of meat product food safety recalls, including losses accrued by upstream and downstream market participants (2), livestock market reactions (3), and retail meat demand impacts (4).

The magnitude of economic losses associated with food recalls has incentivized pre- and post-harvest research, development, and regulation to mitigate impacts. A key question that arises is: What is the optimal amount of investment to reduce future food safety issues? For example, to reduce the occurrence of pathogens in meat products, Hazard Analysis and Critical Control Point (HACCP) regulations were enacted in the United States in 1996. Antle [(5). p. 321] estimated that the annual costs of these regulations “could range from about $500 million to $5 billion (1995 dollars).” Knowing how much consumers devalue products as a result of a food safety breach can help inform the value of improving food safety. However, consumer valuations may differ by the cause and circumstances surrounding food safety recalls.

One way to classify food safety breaches is by their probability and potential severity to human health. Using this method of classification, the United States Department of Agriculture's Food Safety and Inspection Service (USDA-FSIS), which is responsible for inspections and recall notifications for meat, poultry, and egg products, classifies food recalls into three broad classes. Class I (Class II) recalls imply that the potential issue has a reasonable (remote) probability that eating the food will cause human health problems or death, whereas a Class III recall involves a situation in which eating the food will not adversely affect human health. With few exceptions, literature examining the impact of food safety issues has focused on the more frequent and costly Class I recalls such as *E. coli*, Listeria, and Salmonella. Little is known about how consumers react to Class II recalls, which are fewer and tend to have less immediate acute health outcomes.

This research estimates the magnitude of consumer devaluation by food safety class Specifically, we compare consumer valuations for ground beef given either an *E. coli* (Class I) or antibiotic residue (Class II) recall, conditional on the type of retail outlet involved in the recall. From this, we derive consumer willingness to pay for additional private non-government food safety testing. We then empirically test the factors believed to influence consumer ground beef valuation including consumer shopping behavior (6–8), household cooking arrangement (8), organizational trust for food safety information (9), and purchase regret when health can be affected (10, 11).

We focus on *E. coli* and antibiotic residue for several reasons. Antimicrobial residue is an FSIS Class II consumer food safety concern that has recently received considerable public attention. Concerns have centered around allergies to antimicrobial residues, maximum allowable residue levels, and perceived threats to public health through antimicrobial resistance (12). While the number of meat products testing positive for antibiotic residue is low^2^, little is known about how much consumers devalue meat products given an antimicrobial residue recall. *E. coli* is a frequent FSIS Class I consumer food safety concern that has received considerable research and public attention and thus provides an appropriate food safety recall for comparison.

Previous studies examining impacts of food safety recalls due to *E. coli* have used identification strategies that leveraged recall frequency and magnitude. In contrast, only three antibiotic residue FSIS recalls have been issued in the last 5 years, each with relatively small meat volumes recalled. In such situations with infrequent occurrence and small magnitudes, traditional identification fails because of few non-zero values and larger variation. One alternative method, which we use here, is to develop a hypothetical choice experiment to allow for identification and comparison of consumer devaluations given choice attributes. This choice experiment asked a representative sample of U.S. consumers to make repeated choices between three shopping scenarios. Consumers were asked to select the shopping scenario where they would purchase ground beef. Shopping scenario attributes included a potential food safety recall in the previous month (*E. coli* or antibiotic residue), store location (supermarket, club, and convenience), additional private testing, and the price of ground beef. Ground beef valuations and elasticities were derived from estimations which observed consumer purchase decisions across multiple choice sets.

Utility maximization is the behavioral decision rule most often used to obtain consumer valuations, and in particular, in choice experiments involving food purchasing decisions. This rule assumes that consumers evaluate the set of shopping scenarios and then select the one offering the most utility or satisfaction. Despite the popularity of utility maximization, consumers making risky choices where they face (potentially) short or long-term sub-optimal decisions may be subject to regret. One recent alternative framework allows regret to be incorporated as a behavioral decision rule—assuming consumers aim to minimize regret (13). Incorporating regret allows the chosen alternative to depend on the anticipated performance of non-chosen alternatives. This behavioral variation has received increasing attention in transportation, urban planning, environmental economics, and health economics, and when significant potential losses, gains, or policy implications are involved, as is the case with food safety [e.g., (14–20)]. These studies assessed circumstances under which random regret minimization is a more appropriate behavioral assumption than utility maximization. We add to this literature by comparing the relative performance of the Random Utility Model (RUM) and Random Regret Minimization (RRM) under risky decision making in the context of food safety recalls.

We find that 60% of consumers in our sample are better modeled using a regret minimizing framework as opposed to utility maximization. This suggests, in the case of food safety valuation, studies assuming utility maximization might be misclassified and hence lead to incorrect policy recommendations. Our results add to the literature examining differences between random regret and utility maximizing behavioral frameworks by focusing on the context of decisions where there are potential short- and long-run negative impacts on human health. Our work confirms previous findings that a regret minimizing framework may be more appropriate in circumstances where there are actual losses or gains (20). We show that the RRM is the statistically preferred model that generates lower willingness-to-pay estimates and more elastic attribute estimates. Using price as a policy mechanism implies lower price discounts are required to maintain a given level of market share compared to the RUM framework.

The rest of the paper is organized as follows. Section food safety detection, frequency, and impact describes the process of potential food safety issues, frequency and type of recalls, and previous results on the economic impacts of recalls across food safety classes. Section data describes the data and the hypothetical choice experiment used. Section methods presents two simple models to frame consumer choice under two different behavioral assumptions. Section results presents empirical results and simulates potential industry and policy responses. Section discussion and conclusions concludes the article.

## Food Safety Detection, Frequency, and Impact

### Process of Food Safety Detection

The primary objective of a food safety recall is to reduce human health hazards by removing potentially harmful, contaminated, or mislabeled products from the market. Information about products that have been recalled is provided by the U.S. Department of Health and Human Services Food and Drug Administration (FDA) and by USDA-FSIS. The FSIS is responsible for inspecting and regulating meat, poultry and processed egg products produced in Federally inspected plants. All remaining food products are regulated by the FDA.

FSIS works to address potential meat, poultry, and egg safety issues through a five-step process: problem identification, preliminary investigation, recall deliberations, notifications and actions, and recall closure. FSIS identifies potential food safety issues through regular sampling, consumer complaints, epidemiological or laboratory data submitted by public health departments, company self-reporting, and other government agencies. Based on this information, a preliminary investigation can be conducted which includes gathering additional product information and potentially harmed individuals. The objective is to determine whether the alleged product caused, or has the potential to cause, negative health outcomes. With this additional information and analysis in hand, FSIS determines if additional action is warranted. Potential actions include product recall, public health alert, regulatory action, or no action. If a product recall is issued, the recall is classified into one of three safety classes based on relative risk to human health, and the responsible firm is contacted with a request to voluntary recall products.

If the firm agrees to a voluntary recall of potentially harmful products, FSIS notifies the public. The classification of the food safety issue as a human health hazard determines the medium by which FSIS notifies the consumers. A Recall Release is used for Class I and Class II recalls and a Recall Notification Report for Class III recalls. The primary difference between the Recall Notification Report and Recall Release is that the Recall Release is disseminated to public health partners. Regardless of recall classification, all public releases are publicly posted. After public notification of the food safety issue, FSIS works with firms to ensure that they are making reasonable and timely efforts to notify and work with product distributors to remove potentially contaminated products. When a reasonable effort has been made to contact and retrieve potentially contaminated products, FSIS removes the food safety issues from current monitored recalls, and no additional testing or monitoring occurs.

### Type and Frequency of Food Recalls

Food recalls are categorized into one of three classes by their probability to cause human health problems or death. Class I (Class II) implies that there is a reasonable (remote) probability that eating the food will cause human health problems or death whereas Class III recall involves a situation in which eating the food will not adversely affect human health. Examples of a Class I recall includes the presence of Shiga toxin-producing *E. coli* (STECs) in raw ground beef. Class II recall examples include the presence of very small amounts of undeclared allergens associated with milder human reactions (e.g., wheat) or antibiotic residue. Class III recall examples include the presence of non-allergenic products such as excess water in meat products. Thus, recall classes can span across multiple species and processed product formats.

**Figure 1:**
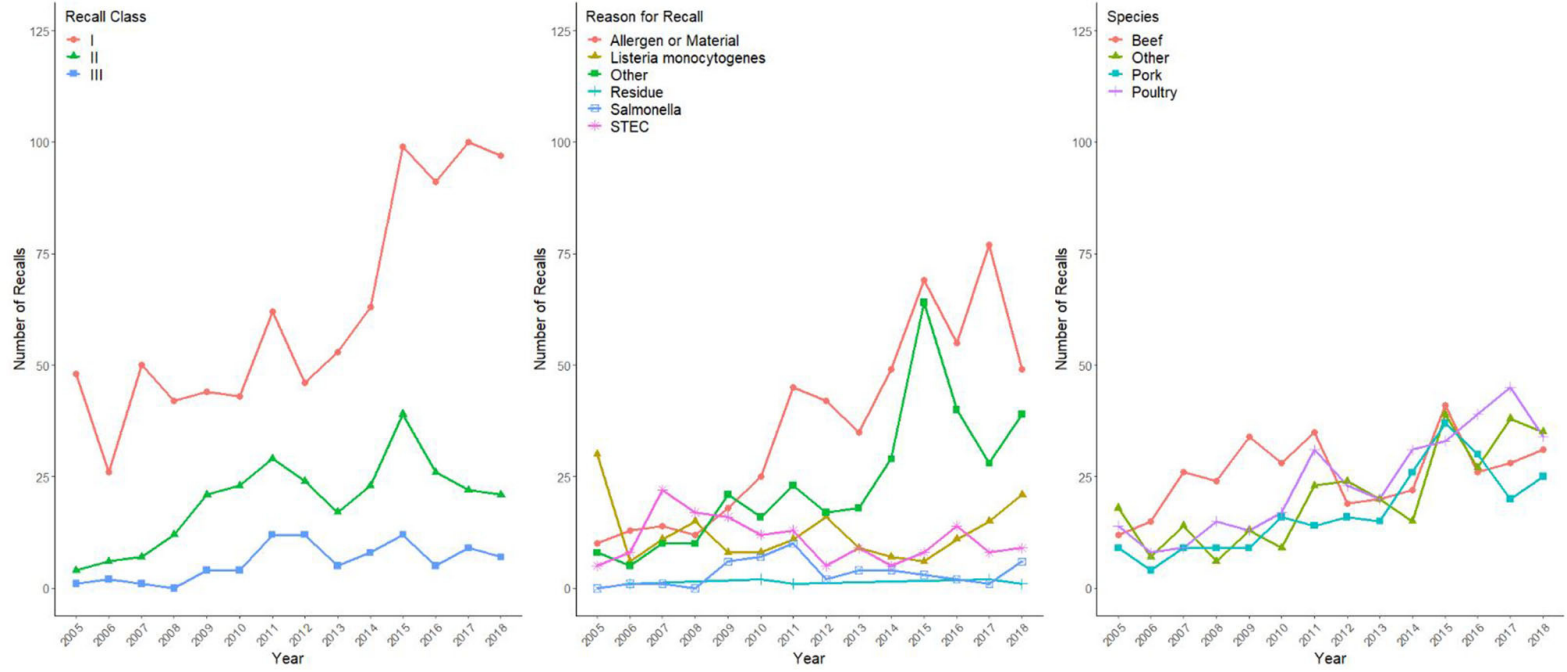
Number of food safety recalls by class, reason, and species (USDA-FSIS 2020).

The far-left chart in Figure 1 displays the number of recalls annually by FSIS safety class. Class I recalls have sharply increased since 2010, whereas Class II and III recalls gradually increased until 2010 but have since leveled off. The distribution of causes for recalls has changed through time. For example, recalls due to *E. coli* contamination were the most common in 2007 but declined year-over-year until 2013 and then stayed constant between 2013 and 2018 (middle chart, Figure 1). Allergens or foreign material recalls were less common in 2005 but sharply increased as the primary reason for food recalls. Although most food issues can span across multiple species and processed product formats, there does not appear to be any difference in frequency across food issues within a given species (far right chart Figure 1). The exception to this is the larger number of beef food safety recalls between 2006 and 2010 compared to other meat and poultry products.

### Impact of Food Recalls

The economic impacts of food safety recalls have been explored extensively. Studies have examined the impact of food safety recalls on company stock prices (21–24), retail meat and livestock prices (2, 3, 25–27), and meat demand (4, 28–30). The impact of recalls is known to have both short- (4) and long-run (31) implications within the species for which the recall occurred and spillover effects into other species. Some studies have focused on the impact of high-volume beef recalls due to *E. coli* [see Moon and Tonsor (2) for a recent example] compared to low occurring Hepatitis A (23) or Bovine Spongiform Encephalopathy (BSE) (26).

Other studies have focused on how mitigating the impacts of food safety recalls affects downstream and upstream participants. Tonsor and Schroeder (32) examined the impacts of adoption of an *E. coli* vaccine at U.S. feedlots. They concluded that feedlots were unlikely to adopt such a vaccine unless compensated to offset the direct costs of adoption. Moon and Tonsor (2) examined price reactions along the beef-cattle supply chain due to an official *E. coli* beef safety recall. Local downstream agents were more likely to be affected financially from an official *E. coli* beef safety recall compared to livestock producers. Thus, implementation of additional risk abatement efforts by local downstream agents, above current practices, were likely to be financially beneficial. Tonsor et al. (9) examined how product characteristics affected consumer perceptions of food safety issues and how trustworthy information sources were viewed. Consumers who placed considerable trust in product credence attributes or in information obtained from health professionals perceived low levels of beef safety risk. Thus, product attributes and information sources can partially mitigate the effects of a food safety recall.

A common thread in past studies is their focus on Class I food safety issues (i.e., where there is a reasonable risk of human health problems or death). This class of recalls tends to be large in volume, more frequent, and highly publicized where human health issues are immediately noticeable (see Figure 1 left chart)^3^. Little work has focused on the impact of Class II food safety recalls, which pose minimal risk to human health. This class of recalls tends to be small in volume, less frequent, and less publicized, even though the subjects of such recalls can potentially affect the long-term health of affected individuals (see Figure 1 left chart). Infrequent occurrence and wide variation in volume in some Class II recalls could lead to biased estimates using commonly used econometric identification strategies.

One way to deal with the issues of recall class heterogeneity is to focus on building demand indices that span across multiple food safety classes (31). Such “food safety indices” do not directly capture the impact by class heterogeneity but rather provide an average or weighted average impact across all classes. If consumer valuations for food safety recalls are a function of the relative probability of illness and death, then the magnitude of impacts should be different across classes. Thus, consumer reactions to and valuations of Class I recalls should be larger in magnitude than those of Class II recalls, and Class II recalls larger than Class III recalls. Likewise, the valuation of Class III recalls should be ~0, given that this class of recalls does not pose any probability of human health problems or death. The relative difference in magnitude between Class I and Class II recalls is uncertain and likely to vary given the food safety issues compared.

It seems obvious that food safety events lower retail prices, since there is a loss of consumer confidence (4) resulting in lower expected utility from consumption and reduced demand. However, no studies have attempted to compare consumer valuations across food safety classes. This study compares one Class I food safety recall (*E. coli*) and one Class II food safety recall (antibiotic residue) in order to understand better consumer perceptions across food safety classes.

## Data

### Sample

The primary objective of this study was addressed by conducting a nationwide online survey of meat-eating shoppers. The survey was developed and pretested by 120 respondents, the majority of whom resided in Kansas. The pretest identified potential issues regarding survey length, questions, and responses. The final survey was then delivered to an online panel of consumers provided by Survey Sampling International (SSI) in the summer of 2017. SSI maintains a list of individuals who “opt-in” to receive and potentially participate in online surveys.

Individuals who received the survey from SSI “opted-in” to complete the survey and respondents who subsequently completed the survey received $4.00 (2017 dollars) from the researchers. Individuals who did not “opt-in” to the survey or exited prior to completion did not receive any monetary compensation. Since our focus was on the individual's valuation of meat products due to a food safety issue, we discarded respondents who did not eat meat^4^. On average, individuals took 25.8 min to complete the survey. In total, 2,696 individuals entered the survey, 2,640 agreed to the associated survey risks, 2,065 completed the survey, of whom 1,994 respondents ate meat. Thus, 1,994 responses were obtained and subsequently analyzed.

Demographic information from the 2010 U.S. Census (33) were used during survey sampling to ensure that survey respondents were representative of U.S. consumers. Table A1 compares survey respondent demographics to 2010 Census values. Survey respondents were slightly more educated, with a higher representation of female participants, and had slightly less income on average. Other demographic characteristics of the sample closely aligned with the 2010 Census. The sample of respondents through SSI may not represent a completely probabilistic sample of the population, which may result in a somewhat less representative sample, given respondents “opt-in” to take the survey. A benefit though is that the results may be more accurate for respondents that opted in, given their potential interest in the survey topic (34).

The survey included questions regarding meat consumption habits, food shopping, and cooking and meal preparation behavior, as well as the usefulness of various organizations for food safety information. Individuals were asked to specify how often they eat a specific meat product using the categories of (i) never, (ii) once a month or less, (iii) two to three times per month, (iv) once per week, and (v) more than once a week. Table A2 summarizes the frequency of meat consumption by meat product. Chicken and beef were the most frequently consumed products. Approximately 57% (45%) of respondents ate chicken (beef) at least once a week. Fish and turkey were the least commonly consumed meat products. Approximately 44% (55%) of individuals ate fish (turkey) once a month or less or not at all.

Individuals were asked what their role was in shopping, the store format where the majority of shopping took place, and their role in cooking. Table A3 summarizes responses to these questions. Across all store formats, ~59% (38.20 + 17.69 + 2.75 = 58.64) of respondents indicated that they did all the food shopping, 26% did the majority of their food shopping, 8% divided food shopping responsibilities, and 8% did the minority of shopping for their household. When choosing a store to purchase food from, ~65% of individuals indicated that they purchased the majority of their food at supermarkets, 30% primarily purchased at club stores, and 5% purchased at “other” stores^5^.

Approximately 61% (40.61 + 18.15 + 2.5 = 61.26) of individuals indicated that they cooked food themselves, 28% cooked together with another household member, and 11% indicated someone else was primarily responsible for cooking. Additional combinations of cooking and shopping habits by store format can be explored using Table A3. For example, ~32% of respondents did all the food shopping, primarily at a supermarket, and were responsible for all of the cooking.

Food safety information sources are known to affect consumer food safety perceptions significantly (9). Respondents were asked to classify 27 information sources as either “helpful,” “somewhat helpful,” or “not helpful” for receiving food safety information. The 27 information sources were allocated into six broad parent groups: government, advocacy groups, producer, store, media, and family and friends^6^. Descriptive statistics are summarized in Table A4. Government and family and friends were viewed as the two most helpful sources of information. Food safety information from the government (family and friends) was considered “helpful” or “somewhat helpful” 58.4% (57.5%). Advocacy groups and stores were viewed as the least helpful for food safety information.

### Stated Choice Experiment

Hypothetical stated choice experiments are a subset of stated choice experiment methods where individuals select what they would do in a hypothetical situation but are not required to take physical action. This methodological subsection is particularly useful when a product or event is infrequently or not observed. These methods have been widely applied in studies of psychology and social behavior (35, 36), public health (37, 38), economics (39, 40), marketing (41, 42), environmental valuation (11), and transportation (43, 44).

**Figure 2:**
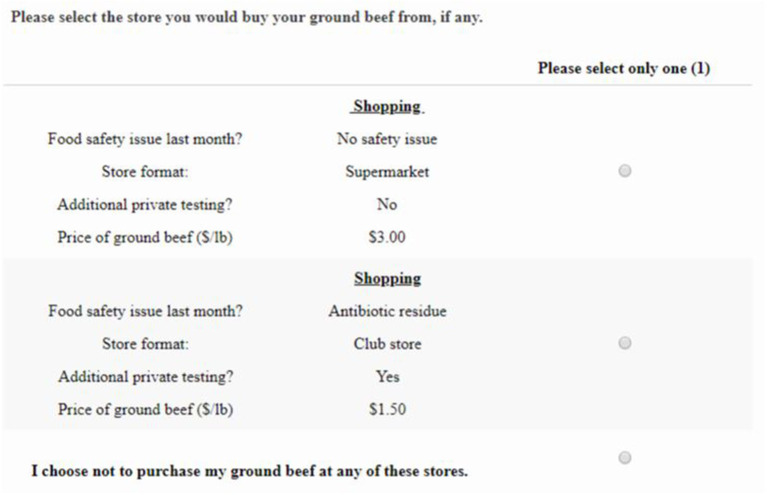
Hypothetical consumer choice scenario example.

A hypothetical stated choice experiment (CE) was created to estimate shopping scenario elasticities and consumer WTP for one pound of ground beef given a hypothetical food safety recall. One concern when using hypothetical stated choice experiments is “hypothetical bias,” the difference between reported/stated and actual action. “Cheap talk” scripts ask individuals to imagine themselves in the situation prior to making a purchase decision and have been shown to reduce hypothetical bias (45). Thus, a cheap talk script was included in the survey prior to stated choice question^7^. Each individual was asked to consider six independent choice sets in which they selected between two shopping scenarios (Shopping A, Shopping B) and a “do not shop” option resulting in three different choice options per choice set (see Figure 2 for an example of one of the choice sets used). Shopping scenario attributes were selected by consulting food safety scientists and a focus group session with U.S. shoppers. The final shopping scenario attributes and attribute levels used are provided in Table 1.

**Table 1:** Contract attributes and levels for stated choice experiments given a hypothetical food safety recall.

**Contract attribute**	**Description**	**Levels**	
		**Level names**	**Number of levels**
Product	Meat product available for individuals to purchase	Ground beef (1 lb.)	1
Time since last food safety issue	Indicates the time since the initial notification from USDA-FSIS to the public about a food safety issue	4 weeks	1
Food safety issue	Indicates the type of food safety issue that has USDA-FSIS has notified the public about, if any	None, *E. coli*, antibiotic residue	3
Private testing	“Yes” indicates the store will provide additional testing to ensure the food safety recall is contained, and “No” otherwise	Yes, no	2
Store format	Indicates the store individuals can purchase their ground beef from	Supermarket, club, convenience	3
Price	Advertised price ($/lb.) for ground beef	$1.50, $2.10, $2.25, $2.40, $3.00	5

Five different prices were used in the choice design. A base ground beef price was established using prices provided by the U.S. Department of Labor's Bureau of Labor Statistics (BLS). The average price of ground beef was $2.25 per pound. Using this as a midpoint, we specified four additional hypothetical prices by adding and subtracting $0.15 and $0.75. Thus, there were five different prices with a spread of $1.50 per pound.

One Class I recall and one Class II recall were selected from a list of potential food safety recalls. *E. coli*, antibiotic residue, and “no food safety issue” were the three food safety issues (attribute levels) selected. *E. coli*, a Class I recall, was selected given the considerable amount of food safety research already conducted and the large investments made by the beef industry and government individuals to improve pre- and post-harvest *E. coli* food safety. Antibiotic residue, a Class II recall, was selected since antimicrobial residue and resistance is a rising concern among consumers. Current beef research is attempting to discover pre- and post-harvest ways to reduce potential antimicrobial residue in meat products. The “no food safety issue” was included to provide a control scenario.

Three store formats were used: supermarket, club, and convenience. About 81 percent of U.S. households purchase much of their food from these types of stores^8^ (46). Among our respondents, ~95% indicated that they purchased food from these types of stores. In the event of a food safety recall, processors work with retail stores to secure the return of potentially harmful products from consumers. When a reasonable effort has been made to contact and retrieve potentially contaminated products, FSIS removes the food safety issue in question from current monitored recalls. No additional testing occurs to ensure that any remaining products are safe to consume. Since no further testing occurs, above the systematic and random sampling for new products shipped to stores, private stores could engage in additional testing to assure customers that food safety issues have been resolved. Thus, regardless of store format, we allow stores to engage in additional/on-site private testing as a measure of consumer quality assurance^9^.

The length of time a food safety recall remains “current/active” varies. The objective of the study is to measure how much consumers discount ground beef after a food safety recall has occurred, but *after* the initial potential threat to human health has decreased, preferably to zero. It is difficult to determine when the minimal potential threat to human health approximates to zero. A food safety recall may be “current/active” for several months or years, but it is unlikely that the threat to human health remains constant through time. One way to proxy whether the potential threat to human health approximates to zero is through an epidemiological outbreak curve which tracks reported health occurrences through time. Using epidemiological outbreak curves from the Centers for Disease Control and Prevention (CDC) for *E. coli*-O157, a time frame of 1 month was selected^10^. Thus, given that a food safety issue potentially happened in the prior month and no immediate health threat is likely present to consumers, the potential discount to ground beef can be viewed as an “intermediate-term” impact.

Given one meat product, one time period, three hypothetical food safety issues, two levels of private testing, three store formats, and five prices, there are 8,100 different possible choice sets ((1 × 1 × 3 × 2 × 3 × 5)^2^) that could be constructed. Thus, we opted to use an orthogonal balanced incomplete (fractional) block design to capture the main and first-order interaction effects. This design is superior to other commonly used designs since it tends to be more reliable, maintains adequate flexibility, accurately captures extreme options, and reduces the burden of excessive questions on respondents (47). PROC OPTEX in SAS was used to develop the incomplete block fractional factorial design, providing 120 random choice sets, which were then grouped into 20 blocks, with each block containing six choice sets that could be used to identify main and interaction effects. The D-efficiency criterion (89.72%) was used to assess the optimal block design efficiency. Each choice set represents a question that a respondent may face in the survey, with each question (choice scenario) asking respondent to choose between three shopping scenarios: “Shopping A,” “Shopping B,” and “Do Not Shop” (an “opt out” option). Each shopping scenario contained attribute information about shopping scenario prices, food safety issues, and level of private testing. Given the large number of choice scenarios, the choice scenarios were blocked into 20 sets of 6 scenarios (or questions). Blocking was done using PROC OPTEX in SAS. Individuals who agreed to participate in the survey were randomly assigned to one of the blocks of six questions (or choice scenarios) followed by questions concerning their consumption and cooking habits, as well as access and preference for food safety information. Prior to answering the set of choice questions, participants were presented with a cheap talk script. The six choice questions (sets) were randomly ordered to avoid any potential order bias. After completing the stated choice experiment and subsequent questions, participants were asked some follow-up demographic questions, after which they then exited the survey.

## Methods

### Random Utility Maximization Model

The Random Utility Maximization framework (RUM) assumes individuals are utility maximizers, where utility is derived from the attributes of the product or good being consumed (48). Given that the researcher only observes the choice of the consumer, it is assumed that the individual's utility function is represented as $U_{nit}=\beta^{\prime }{\text{X}}_{nit}+\;\varepsilon_{nit}\;$, where *X* is a vector of attributes observed for individual *n* (1, …, *N*) when choosing alternative *i* (1, …, *I*) in choice set *t* (1, …, *T*), β is a vector of parameters to be estimated, and ε is the unobserved part of utility. Given that ε is unobserved, we treat ε as a random variable. Following Train (49), we assume ε is distributed Extreme Value Type I. Train (49) explains that this distributional assumption does not differ substantially from the normal distribution, but does allow for more aberrant behavior given it has fatter tails. In addition, this distributional assumption allows for a closed-from solution for the choice probabilities of interest in this study. The probability of an individual choosing alternative *i* over alternative *j* in choice set *t* is given by the Random Utility Multinomial Logit (RUM-MNL) function:

(1) $$\begin{array}{l} P_{nit}=\frac{exp\left(V_{nit}\right)}{\sum_{j=1}^{J}\exp\left(V_{njt}^{\prime }\right)} \end{array}$$

where $V_{nit}=\;\beta^{\prime }{\text{X}}_{nit}$.

The RUM-MNL assumes that all individuals have similar views on food safety recall attributes. It is likely that these views vary across individuals and that groups of individuals have similar tastes and preferences. We allow for this type of heterogeneity using a latent class model in which attribute estimates are assumed to be similar within groups/classes but different across groups/classes. The approach and number of classes to include was determined using a combination of the Akaike information criterion [AIC; (50)], adjusted Bayesian information criterion [BIC; (51)], and relevant class sizes consisting of at least 20% of the individuals (52, 53). Thus, we modify Equation 1 to allow estimation of classes, *C*, given as:

(2) $$\begin{array}{l} P_{nit}=\sum_{c=1}^{C}P_{nc}\frac{exp\left(V_{nit}\right)}{\sum_{j=1}^{J}\exp\left(V_{njt}^{\prime }\right)} \end{array}$$

where *P*_*nc*_ is the probability of individual *n* being in class *c*, *V*_*nit*_ is the same as described above expect now the parameters are class specific (i.e., β_*c*_). *P*_*nc*_ is assumed to be a function of individual-specific characteristics that describe the characteristics of the group. That is: $P_{nc}=\frac{exp\left(\gamma_{c}^{\prime }{\text{Z}}_{n}\right)}{\overset{R}{\underset{r=1}{\sum }}\exp\left(\gamma_{r}^{\prime }{\text{Z}}_{n}\right)}$, where **γ**_*c*_ is a vector of class parameters to be estimated and **Z**_*n*_ is a vector of individual specific covariates. We assume class membership is determined by trust in informational sources, buying behavior, and cooking behavior (6–8).

### Random Regret Minimization Model

Nearly all hypothetical choice experiments have been based on the behavioral assumption of utility maximization. Decision rules within this framework aim to model the marginal (dis)utility attached to alternative-specific attributes. Despite the popularity of the utility maximization framework, various attempts have been made to relax its underlying premises which at times lack behavioral realism. One alternative framework, the Random Regret Minimization (RRM), allows a chosen alternative to depend on the anticipated performance of non-chosen alternatives. This behavioral variation in the decision rule implies that the selection of a specific alternative is affected by the relative (non)performance of one or more non-chosen alternatives. If one or more of the alternatives perform better than the chosen alternative, then this choice would cause an individual “regret.” Thus, the behavioral assumption assumes that an individual seeks to minimize anticipated regret rather than maximize utility from a given choice (54).

Empirical evidence supports this behavioral modification of regret compared to utility maximization. For example, microeconomics and psychology both find that regret is an important determinant in choice behavior (55–58). Regret is particularly present when making risky choices where individuals must absorb (potentially) short or long-term sub-optimal decisions. Likewise, there is evidence that modeling regret, compared to utility maximization, is more appropriate when there are significant potential losses or gains involved (20).

The forgoing discussion about model estimation in this context can now be modified to show how the Random Regret Minimization model differs from the commonly accepted assumption of Random Utility Maximization. The RUM aims to capture the marginal (dis)utility attached to alternative-specific attributes. The RRM assumes individuals minimize the sum of binary regrets between alternatives. Thus, for a given alternative *i*, regret occurs when it is outperformed by alternative *j* on attribute *m*. Letting there be *m* (1, …, *M*) attributes, regret can be modeled as:

(3) $$\begin{array}{l} RR_{i}=R_{i}+\epsilon_{i}\\ =\sum_{j\neq i}\sum_{m=1}^{M}\ln\left(1+\exp\left(\left[\delta_{m}\times \left(x_{jm}-x_{im}\right)\right]\right)\right)+\;\epsilon_{i} \end{array}$$

where *RR*_*i*_ is the total regret associated with alternative *i*, *R*_*i*_ is the observed regret associated with alternative *i*, ϵ_*i*_ is the unobserved regret associated with alternative *i*, δ_*m*_ is an estimated parameter associated with attribute *x*_*m*_, and *x*_*im*_ and *x*_*jm*_ are values associated with *x*_*m*_ for considered alternative *i* and alternative *j*. Regret for alternative *i* approaches zero with respect to attribute *m* when the difference between *x*_*jm*_ and *x*_*im*_ is at least <0. The Logsum formulation of attribute-level regret in Equation 3 acts to smooth the regret-function and allows for an approximation that is differentiable and globally concave (59).

Similar to the RUM framework, different models arise given different distributional assumptions for ϵ_*i*_. If the negative of the errors (ϵ_*i*_) is distributed as Extreme Value Type I, the choice probabilities gives rise to the Random Regret Minimization Multinomial Logit (RRM-MNL) model, where the choice probability can be written as:

(4) $$\begin{array}{l} P_{nit}=\frac{exp\left(-R_{nit}\right)}{\sum_{j=1}^{J}\exp\left(R_{njt}^{\prime }\right)} \end{array}$$

where *R*_*nit*_ is the random regret for individual *n* choosing alternative *i* in choice set *t*. It is important to note that Equation 4 is maximizing the negative of random regret, which is mathematically equivalent to the minimization of random regret (20).

We believe that the behavioral assumption of the model should not alter the presence of individual preference heterogeneity. Likewise, we believe that these taste preferences are still different across but similar within groups of consumers. The decision rule to identify classes/groups of individuals is the same as the rule used under the RUM-MNL framework. The latent class model is derived by modifying Equation 4 as follows:

(5) $$\begin{array}{l} P_{nit}=\overset{C}{\underset{c=1}{\sum }}PR_{nc}\frac{exp\left(-R_{nit}\right)}{\sum_{j=1}^{J}\exp\left(R_{njt}^{\prime }\right)} \end{array}$$

where *PR*_*nc*_ is the probability of individual *n* being in class *c*, *R*_*nit*_ is the same as described above expect now with class-specific parameters. The specification of *PR*_*nc*_ follows that of the RUM-MNL model presented in the previous subsection.

### Hybrid Model: Random Utility Plus Random Regret

It is unlikely that every individual or group of individuals views choice decisions under a regret minimizing or utility maximizing framework. Some individuals may aim to maximize utility while others may minimize regret. In our context of making decisions about potential ground beef decisions after a potential food safety issue some combination of utility maximization and regret minimization seems particularly important. Previous research suggests that there is significant variation in how individuals view the cost or likelihood of a food risk occurring (60). Proper categorization of individuals is important since managerial and policy decisions are likely to differ substantially between these groups. Whether groups of individuals exist, the relative size of said groups, and the differences in managerial and policy implications arising from these two groups can be empirically tested.

We test this hypothesis by using a latent class model where some classes/groups are constrained to be utility maximizing and others regret minimizing. This hybrid model specifies that some choice probabilities follow Equation 2 under the random utility framework while other choice probabilities follow Equation 5 under the random regret minimization framework. Hess and Stathopoulos (61) describe in detail how this type of model is specified and estimated using the framework provided for the random utility and random regret models. The optimal number of classes under each behavioral framework is determined using the latent class decision rule described for the other models previously discussed. We more deeply explore which socioeconomic covariates characterizing individual *n* are likely to be related to risk-minimizing or utility-maximizing behavior for food safety recalls using this hybrid framework.

### Data Quality Checks and Methods of Comparison

Survey length and monetary compensation for survey completion were a potential concern for response quality, and thereby estimation (62, 63). Researchers traditionally overcome these issues by asking engagement or inattention questions randomly throughout the survey. Individuals who fail inattention questions are generally removed prior to estimation since they pose a significant threat to data quality and can lead to significant violations to axioms of revealed preferences (63, 64). Recent research suggests that inattentive individuals can be captured through a latent class model where one class is restricted to zero known as the “random response share” [RRS; (65)].

We determined where there are inattentive respondents in our survey by estimating Equations 1 and 4 and then statistically testing model fit against Equations 2 and 5 with two classes where one class is restricted to zero (65). All models were estimated in NLOGIT6. Latent class models under both behavioral frameworks identified 31% of respondents as producing random responses highlighting the importance of accounting for this class of individuals. A log likelihood test determined that by accounting for identified non-attentive individuals, model fit significantly improved. Thus, all models reported in subsequent tables were estimated using a latent class model where one class was restricted to zero to capture RRS.

## Results

### Multinomial Logit Estimation Under Both Behavioral Frameworks

Table 2 presents estimates under two different behavioral assumptions: random utility maximization (RUM) and random regret minimization (RRM)^11^. The McFadden Pseudo *R*^2^ values indicates a relatively decent fit to the data. Although the AIC and log likelihood values appear similar, suggesting statistically similarity between models, the traditional log likelihood tests can only be used to compare statistical differences between nested models. Since the RUM and RRM models are non-nested, traditional log-likelihood tests are inappropriate. The Vuong test (66) is one non-nested testing procedure that is applicable when there are two or more choice alternatives and the models may be observationally equivalent. The null hypothesis for the Vuong test is that the two behavioral frameworks are equally “close” or are representative of the true data generating process. The alternative hypothesis is that only one of the behavioral frameworks is closer or more representative of the true data generating process. However, the test cannot determine if the “closer” framework *is* the true data generating process. A Vuong statistic < -2 favors the alternative hypothesis, a value > +2 favors the null hypothesis, and a value between −2 and +2 is inconclusive. Using model information from Table 2, we calculate the Vuong test statistic. The test statistic is −4.12 implying that that the RRM (alternative model) is closer to the true data generating process than the RUM. Thus, the RRM is preferred in comparison to the RUM.

**Table 2:** Latent class model estimation with random response share under alternative behavioral assumptions.

	**Random utility maximization (RUM)**			**Random regret minimization (RRM)**		
	**Mean**	**SE**	**WTP ($/lb.)**	**Mean**	**SE**	**WTP ($/lb.)**
Constant	0.587^**^	0.069		0.933^**^	0.137	
Price	−0.354^**^	0.032		−0.521^**^	0.053	
*E. coli*	−2.939^**^	0.101	−8.30	−4.393^**^	0.123	−6.31
Residue	−1.736^**^	0.045	−4.90	−2.816^**^	0.072	−3.77
Club store	0.732^**^	0.049	2.07	1.213^**^	0.073	2.60
Supermarket	1.069^**^	0.049	3.02	1.706^**^	0.072	3.87
Private testing	0.506^**^	0.036	1.43	0.801^**^	0.054	1.66
RRS	0.307^**^			0.309^**^		
McFadden *R*^2^	0.235			0.233		
LL	−10,045			−10,072		
AIC	20,106			20,161		
N	11,9641			11,964		

** *indicates significance at the 0.01 level*.

All parameters in Table 2 have the expected sign and are statistically significant at the 0.01 level. While the models are statistically similar to each other and coefficient signs are all the same, the direct comparison of magnitudes of the coefficients across behavioral frameworks is not meaningful (67). Where RUM parameters signify the contribution of an attribute to an alternative's utility, RRM parameters signify the potential contribution of an attribute to the regret associated with an alternative conditional on non-chosen alternatives (20). Even though the magnitude of coefficients between the RUM and RRM frameworks cannot be directly compared, the relative odd ratios between parameters within the same framework is meaningful. Thus, we calculate the odd ratios between parameter estimates.

Under both frameworks, the likelihood of purchasing ground beef given an *E. coli* (Class I) recall is greater (i.e., more negative) than a food recall due to antibiotic residue (Class II). Both food recall estimates are negative indicating the odds of purchasing ground beef given either food safety recall decreases when either food safety issue is present. Respondents have lower odds to purchase ground beef given an *E. coli* recall relative to antibiotic residue in the past month. The odds of purchasing ground beef after an antibiotic residue recall compared to *E. coli* is 0.21–0.30, suggesting that respondents viewed *E. coli* as a greater threat to their health and therefore avoided purchases with greater frequency. We observe larger odds ratios under the RRM framework, suggesting that under risky food decisions, regret may be a statistically more important behavioral component than marginal utility. This supports the idea from Ajewole et al. (60) that individuals view food safety risks having an immediate impact on human health as more problematic than those with delayed impacts. Likewise, these results support our previous hypothesis that consumer valuations for food safety recalls are an increasing function of the relative probability of human illness and death. Thus, consumer reactions/valuations to Class I recalls (i.e., *E. coli*) should be larger in magnitude than Class II recalls (i.e., antibiotic residue).

The odds of purchasing ground beef at a supermarket is ~40–63% greater than purchasing it at a club store (*e*^1.069^/*e*^0.732^ = 1.40). Under the RRM framework, individuals have greater odds of purchasing ground beef at supermarkets. This result confirms the findings from Stern et al. (46), that in 2012, individuals purchased more food from supermarkets than from club stores. *Club Store* and *Supermarket* estimates are positive, indicating that individuals viewed both store formats as more viable locations to purchase ground beef when compared to convenience stores. If stores were to adopt private food safety testing, the odds of purchasing ground beef would increase by ~65–120% depending on the behavioral framework.

The differences in odd ratios between behavioral frameworks has potential important managerial and policy implications targeted at reducing food safety issues across different health classifications. Compared to the RUM framework, the RRM estimates suggest that individuals view *E. coli* as being more harmful than antibiotic residue. In addition, individuals have greater odds of purchasing ground beef if there were private store testing. Policies based on estimates from RRM would imply conducting less research and development for pre- and post-harvest practices to reduce antibiotic residue in meat products and a greater need for private store testing.

### Elasticities and Willingness-To-Pay Estimates

Direct interpretation of estimated coefficients from the behavioral frameworks in Table 2 is not straightforward. One alternative is to calculate and compare direct choice elasticities. Elasticities allow comparison across attributes within a behavioral framework, but interpretations of elasticities within a given framework may differ. Under the RUM framework, elasticities depend only on the performance or choice probability of the specific alternative. However, given the behavioral premise of the RRM framework that regret for a given alternative is based on the relative performance of non-chosen alternatives, interpretation of elasticities is slightly different. Namely, changes in the alternative attribute depend on the relative performance of all the alternatives in the choice task being assessed.

Our experimental design was an unlabeled choice in which individuals selected between shopping experiences. Calculating an elasticity for each store would not yield additional information since there is no intrinsic value in the elasticity differences between “Shopping A” and “Shopping B” for a given attribute. Thus, following Thiene et al. (15), we calculate and report the mean difference between “Shopping A” and “Shopping B.” Due to these differences in the interpretation of elasticities between frameworks, it is more appropriate to calculate an elasticity ratio. We calculate this as the ratio between the absolute value of the mean difference of the RUM framework divided by the absolute value of the mean difference of the RRM framework. A ratio >1 indicates an attribute is more elastic under the RUM framework. As the ratio approaches zero, it implies that the RRM elasticity is more elastic.

Table 3 reports the direct elasticities for shopping scenarios A and B, the difference between these scenarios, and the elasticity ratio. All elasticities in both frameworks are significant at the 0.05 level, all attributes have the expected signs, and attributes are relatively small. Five of the six attributes become more elastic under the RRM framework than in the RUM framework. Most attributes were significantly more elastic under the RRM framework compared to the RUM framework. The only noticeable difference is that of *Private Testing* which tended to be more elastic under the RUM framework.

**Table 3:** Direct elasticities and relative differences for latent class model with random response share.

**Attribute**	**Random utility maximization (RUM)**			**Random regret minimization (RRM)**			**Elasticity ratio abs(RUM)/abs(RRM)**
	**St1**	**St2**	**St1-St2**	**St1**	**St2**	**St1-St2**	**St1-St2**
Price	−0.340^**^	−0.340^**^	0.000	−2.140^**^	−2.017^**^	−0.123	0.001
*E. coli*	−0.305^**^	−0.303^**^	−0.002	−4.320^**^	−4.381^**^	0.061	0.026
Residue	−0.349^**^	−0.355^**^	0.006	−1.516^**^	−1.260^**^	−0.256	0.025
Club store	0.118^**^	0.133^**^	−0.015	0.584^**^	0.573^**^	0.011	1.343
Supermarket	0.172^**^	0.173^**^	−0.001	0.766^**^	0.723^**^	0.043	0.021
Private testing	0.111^**^	0.120^**^	−0.009	0.554^**^	0.568^**^	−0.014	0.615

** *indicates significance at the 0.01 level*.

Under both frameworks, choices made in *Supermarkets* are more elastic than those made in *Club Stores*. This result may arise from consumers who shop at supermarkets, buy small amounts of select items. Thus, these consumers are likely to be more price sensitive, whereas club store consumers generally buy bulk amounts of products. Under the RUM framework, Residue (Class II) is more elastic than *E. coli* (Class I), but the opposite is true under the RRM framework. This shows that using an appropriate behavioral framework is important in determining consumer attitudes toward food safety.

Willingness-to-pay (WTP) is another method to examine and compare alternatives. WTP calculations are conditional on the behavioral framework used and have been frequently used to determine valuation of environmental amenities [e.g., (68)], food attributes [e.g., (69)], and reductions in risk [e.g., (70)]. Under the RUM framework in Equation 1 where all the non-cost and cost attributes enter the utility function, WTP estimates can be estimated as:

(6) $$\begin{array}{l} WTP_{RUM}=\;-\frac{\beta_{t}}{\beta_{c}} \end{array}$$

where *t* represents any non-cost attribute and *c* is the cost attribute. Thus, the WTP is the ratio of attribute coefficients. The behavioral assumptions under the RRM framework imply a different WTP formulation. Using Equation 3, where all the non-cost and cost alternatives enter the regret function, Chorus (67) shows that the WTP can be estimated as:

(7) $$\begin{array}{l} WTP_{RRM}=-\frac{\beta_{t}}{\beta_{c}}\frac{\sum_{j\neq i}\left\{\frac{1}{1+\frac{1}{\exp\left[\beta_{t}\left(x_{ji}-x_{it}\right)\right]}}\right\}}{\sum_{j\neq i}\left\{\frac{1}{1+\frac{1}{\exp\left[\beta_{c}\left(x_{jc}-x_{ic}\right)\right]}}\right\}} \end{array}$$

where *j, i* are attributes, *t* is the non-cost alternative, and *c* is the cost attribute. Here, it is relatively straightforward to see how the *WTP*_*RRM*_ is a weighted version of the *WTP*_*RUM*_, where weights are determined by the relative performance of non-chosen alternatives.

WTP estimates ($/lb.) are reported in Table 2 for the RUM and RRM frameworks. Under the RUM framework, individuals were willing to pay an additional $1.43 per lb. of ground beef if a store engaged in additional food safety testing. Consumers were willing to pay approximately a dollar more to purchase ground beef in Supermarkets compared to club stores. Consumers discounted ground beef by ~$8 ($5) per pound if an *E. coli* (antibiotic residue) recall occurred in the prior month. Given that the discount was larger than the average price of ground beef, this result implies that even though consumers may be willing to shop they would not likely purchase ground beef after these types of events.

The RRM estimates of WTP are much different from RUM estimates. The discount from an *E. coli* recall on ground beef was ~$6 per pound, compared with $4 per pound for an antibiotic residue recall. These estimates are approximately $2 and $1 lower than corresponding estimates under the RUM framework, respectively. Differing WTP estimates were found for other attributes as well. The lower WTP estimates using the RRM framework may indicate a “regret adjusted” WTP, whereas WTP under RUM encompass a “risk/regret” premium. That is, risk-averse consumers may prefer to minimize risk (regret) rather than maximize utility *in situations* dealing with potentially hazardous food risks. The differences found between the behavioral frameworks emphasizes the need to identify correctly individuals purchasing behavior intentions in order to make viable managerial and policy decisions.

### Comparison of Latent Class Models and a Combined Framework

Table 4 displays the results for the latent class models estimated under the RUM, RRM, and RUM+RRM frameworks. Three optimal groups of individuals were identified under each framework using the decision rule to identify major classes based on AIC and BIC: two RUM groups under RUM framework, two RRM groups under RRM framework, and one RUM + one RRM group under the hybrid framework. Our previous results suggest that correctly identifying and categorizing individuals as either RUM or RRM. We now attempt to differentiate between consumer behavior related to risky choice-making based on respondent personal characteristics. As such, a combination of organizational trust, shopping habits, and cooking arrangements were used to attempt to understand the sociodemographic makeup of each class. These factors were selected based on strong predictors of food purchase decisions identified by prior studies of food safety and marketing [e.g., (71–74)].

**Table 4:** Latent class estimation with random response share under alternative specifications.

	**Random utility maximization (RUM)**				**Random regret minimization (RRM)**				**Hybrid model (RUM + RRM)**			
	**Class 1**		**Class 2**		**Class 1**		**Class 2**		**Class 1**—**RUM**		**Class 2**—**RRM**	
	**Mean**	**SE**	**Mean**	**SE**	**Mean**	**SE**	**Mean**	**SE**	**Mean**	**SE**	**Mean**	**SE**
**Choice Attributes**												
Constant	0.601^*^	0.149	2.465^*^	0.191	0.413^*^	0.079	1.315^*^	0.097	2.250^*^	0.187	0.423^*^	0.081
Price	−0.498^*^	0.060	−0.311^*^	0.064	−0.349^*^	0.037	−0.198^*^	0.038	−0.324^*^	0.064	−0.360^*^	0.038
*E. coli*	−4.281^*^	0.130	−1.650^**^	0.135	−2.886^*^	0.116	−1.024^*^	0.080	−1.833^*^	0.140	−2.947^*^	0.125
Residue	−3.117^*^	0.092	−0.882^*^	0.102	−1.970^*^	0.064	−0.567^*^	0.063	−1.011^*^	0.107	−2.032^*^	0.067
Club store	1.079^*^	0.079	0.720^*^	0.087	0.636^*^	0.053	0.530^*^	0.065	0.835^*^	0.090	0.628^*^	0.055
Supermarket	1.675^*^	0.076	0.836^*^	0.085	1.049^*^	0.053	0.628^*^	0.064	0.962^*^	0.089	1.059^*^	0.054
Private testing	0.708^*^	0.059	0.619^*^	0.066	0.447^*^	0.039	0.440^*^	0.048	0.676^*^	0.068	0.447^*^	0.040
**Class Attributes**												
Constant	1.825^*^	0.656	0.835	0.733	1.694^*^	0.587	0.711	0.675	0.607	0.657	1.591^*^	0.568
Trust—government	0.257^*^	0.064	0.199^*^	0.071	0.257^*^	0.064	0.204^*^	0.071	0.212^*^	0.067	0.257^*^	0.060
Trust—advocacy	−0.488	0.256	−0.625^**^	0.280	−0.435	0.243	−0.584^**^	0.268	−0.526^**^	0.253	−0.381	0.228
Trust—producer	−0.047	0.118	0.075	0.128	−0.075	0.112	0.043	0.124	0.028	0.118	−0.088	0.106
Trust—store	−0.230^**^	0.096	−0.151	0.105	−0.244^*^	0.093	−0.165	0.103	−0.194^**^	0.096	−0.258^*^	0.087
Trust—media	−0.003	0.045	0.050	0.049	−0.013	0.043	0.039	0.047	0.031	0.044	−0.0186	0.040
Trust—family	0.123	0.090	0.108	0.100	0.114	0.088	0.104	0.099	0.118	0.094	0.119	0.082
Buy—all	−0.368	0.624	−0.259	0.680	−0.284	0.556	−0.210	0.616	−0.282	0.601	−0.333	0.541
Buy—majority	−0.528	0.636	−0.434	0.696	−0.440	0.566	−0.334	0.632	−0.342	0.613	−0.462	0.548
Buy—equal	−0.696	0.719	−0.532	0.796	−0.718	0.642	−0.534	0.730	−0.505	0.711	−0.729	0.624
Cook—I	0.269	0.431	0.771	0.498	0.226	0.424	0.788	0.496	0.838	0.470	0.230	0.399
Cook—we	0.776	0.453	1.029^**^	0.517	0.683	0.427	0.968	0.498	1.034^**^	0.476	0.697	0.406
Class Probability	0.627^*^	0.015	0.296^*^	0.015	0.617^*^	0.016	0.302^*^	0.015	0.310^*^	0.015	0.603^*^	0.016
**Model Performance**												
RRS	0.076^*^				0.080^*^				0.085^*^			
McFadden *R*^2^	0.285				0.282				0.282			
LL	−9,391				−9,436				−9,429			
AIC	18,859				18,949				18,935			
*N*	11,964				11,964				11,964			

*, ** *indicates significance at 0.05 and 0.01 level respective*.

Approximately 8% of individuals were identified as producing random responses across all models. This suggests regardless of the underlying behavioral framework, the random response method (65) was able to identify a similar magnitude of individuals who were inattentive. The McFadden Pseudo R^2^ indicates a relatively good fit to the data for each of the models. We compared model fit across frameworks using the Vuong test. We calculated the Vuong test statistic for each binary comparison. In total, we have three test statistics, RUM vs. RRM, RUM vs. RUM+RRM, and RRM vs. RUM+RRM. We find RUM was inferior to either RRM or RUM + RRM but both RRM and RUM+RRM models have similar statistical fit (−4.12, −3.22, and 1.70, respectively). This suggests that the behavioral assumptions have an impact upon model performance.

Estimated coefficients from choice attributes across all the frameworks are significant at the 0.05 level and have the expected signs. A negative price sign indicates that as price goes up the odds of purchasing ground beef decreases consistent with economic theory. Negative coefficients on *E. coli* and Residue are likewise consistent with economic and food safety theory, indicating that when a food safety recall occurs, the odds that a consumer purchased ground beef decreased. One question specific to this paper is the relative magnitude of an *E. coli* (Class I) recall compared to an antibiotic residue (Class II) recall. Table 4 suggests that the odds of purchasing ground beef after an *E. coli* recall were lower than after an antibiotic residue recall across all frameworks and groups of individuals.

As previously mentioned, the relative magnitude of coefficients across behavioral frameworks cannot be compared, but the relative magnitude of coefficients across models can (75). Under each behavioral framework, there is one class/group of consumers which is relatively less likely to purchase ground beef in the event of an *E. coli* recall. For example, under the RUM framework, the coefficient ratio of *E. coli* to Residue is 1.87 and 1.37 for individuals identified in class two and class one respectively—or about 36% smaller. Similar comparisons can be made for the RRM and RUM+RRM frameworks.

*Club Store* and *Supermarket* both have positive coefficients, indicating that consumers view these store formats as viable locations to purchase ground beef. However, the larger coefficient on *Supermarket* compared to *Club Store* across all behavioral frameworks and classes of individuals suggests that the odds of purchasing ground beef are greater at a supermarket than at a club store. The coefficient for P*rivate Testing* is likewise positive, indicating that if stores were to engage in private testing of meat products, the odds of consumers purchasing ground beef from any store format would increase.

### Class Specific Sociodemographic Attributes

The individual characteristics making up each consumer class (or segment) indicate that across behavioral frameworks, specific types of trust play an important role in ground beef purchases. Trust in each class is measured as the difference between the number of sources consumers found to be very helpful vs. not helpful. Our hypothesis is that as trust increases, so, too, will some individuals' odds to purchase ground beef. Across all classes of individuals and behavioral frameworks, as governmental trust increased, so, too, did the odds of buying ground beef. These results support the conjecture that if consumers trust the government, or specifically in the case of recall information from FSIS, then the odds of purchasing ground beef after a food recall in the previous month would increase. This likely indicates that trust from other non-USDA governmental branches can have trust “spillover” effects on ground beef purchasing.

Trust in advocacy groups never increased ground beef consumption, but for select groups of consumers, the odds of purchasing ground beef decreased. This decrease in odds of purchasing ground beef is likely due to advocacy groups being generally oriented toward exposing or promoting negative information about ground beef consumption. Surprisingly, as trust increased in stores, the odds of purchasing ground beef decreased. There appears to be two conflicting results. First, Table A4 suggests that a large majority of individuals do not view stores as a helpful source of food safety information. Second, Table 2 suggests that consumers are willing to pay a premium for ground beef if stores engaged in private testing. We are unsure of why store trust would decrease ground beef purchasing, but we do note that the relative magnitude of *Private Testing* is larger for classes of individuals where store trust is low. Trust in Producers, Media, and *Family* all have no statistical impact on identifying class membership based on ground beef purchasing behavior. Thus, the increasing presence of producer promotion programs may do little to increase the odds of purchasing ground beef given a food safety recall.

Food purchasing and cooking behavior were also examined as potential individual attributes which could explain class identification. These two groups of characteristics have been used in studies to help explain food purchase decisions (76). We find that the self-identified proportion of groceries purchased by consumers has little impact on the odds of purchasing ground beef. One plausible explanation for this is that home meal menus are jointly created and thus ground beef is on the shopping list regardless of who purchases the food. Evidence for this hypothesis should appear in the household cooking arrangement. If menus are jointly created, then cooking jointly should be significant. We find that only the *Cook-We* arrangement significantly increased the odds of ground beef purchases, but only for a select subset of individuals—generally, the class with a smaller class probability. Thus, this hypothesis holds but is not true across all consumers. Other cooking did not significantly explain class/group membership.

### Classes Across Behavioral Frameworks

Comparing the attributes that make up each class within and across behavioral frameworks reveals persistent consumer behavior. Generally, there are two broad groups of consumers across all frameworks. The first group is identified as viewing an *E. coli* (Class I) or antibiotic residue (Class II) as similar, preferring to purchase ground beef at supermarkets and trusting government sources, while distrusting stores. This group consists of 63% of respondents. This group is seen in the RUM-class1, RRM-class2, and RUM+RRM-class2 classes. The second group consisted of consumers who view *E. coli* as a more problematic food safety issue than antibiotic residue, trusted government, distrusted advocacy groups, and where cooking was shared among household adults. This group consists of 30% of respondents. This group is seen in the classes RUM-class2, RRM-class2, and RUM+RRM-class1.

Results up to this point show overwhelming support for the need to correctly identify individuals who are either utility-maximizing or regret-minimizing. Columns 10–13 of Table 4 display the estimates for the RUM+RRM model, where one class is restricted to RUM and the other is RRM [e.g., (61)]. The first class, categorized as RUM, consisted of 31% of the population. The second class, categorized as RRM, consisted of 60% of the population. These individuals trust government sources and distrust stores as a source for food safety information.

These consumers account for the performance of non-chosen alternatives in making choices and attempt to minimize regret in purchasing potentially risky foods. This appears to align well with intuition in the context of food safety recalls. For example, consumers view all food as inherently “safe” when going to the store to purchase food. Under a situation where a food safety recall has occurred in the previous month, consumers may perceive that the likelihood of purchasing “safe” or “healthy” food is <1. This could cause them to become risk averse and thus avoid making a food purchasing decision in the event the food safety issue has not been sufficiently resolved. The consumer may wish to minimize the regret of making an incorrect food purchase decision and avoid any potential immediate or long-term effects due to, for example, “food poisoning” ^12^. The fact that the two subgroups appear across different behavioral frameworks emphasizes the point that correctly modeling consumer decision making is important to the analysis and evaluation of policy options. Under stricter assumptions, one could say that 60% of the individuals in the RUM model were “misclassified,” as they do not aim to maximize utility when purchasing ground beef but rather minimize potential regret when making their decision. A corollary argument could be made for 30% of individuals in the RRM framework. However, a combination of the two classes appears to allow for better identification. This is particularly important given that both models are statistically similar and, as we will see in the next section, different conclusions for industry and governmental policy could be drawn.

### Price as a Policy Mechanism

We now explore how differences across behavioral frameworks give rise to different implications for government policy and industry responses to a given food safety recall. Price is the primary mechanism that stores can use to incentivize ground beef purchases. One reason stores discount price after a food safety recall is to encourage ground beef purchases to offset an individual's “food safety risk premium.”

We explore how different price discounts affect the probabilities of various ground beef purchasing choices. We do so by using the estimates from Table 4 to calculate shifts in consumer choice probabilities and total changes given either a 10 or 50% price discount. Since we have an unlabeled hypothetical choice experiment, we allow the discount to occur separately in either “Shopping A” or “Shopping B” and then average the change in choice probabilities. We call the shopping experience where the discount occurred as *Discounted*, the shopping experience where the discount was absent *Non-discounted*, and the choose-not-to-shop option *No purchase*.

Table 5, panel (a), reports these findings across the three different behavioral models estimated in the paper (RUM, RRM and RUM+RRM). Column one is the different price discounts and discount scenarios. Columns two, four, and six [labeled “Change in choice probability (%)”] are the average changes in choice probabilities for the three behavioral models for each respective shopping scenario. Columns three, five, and seven [labeled “Total change (%)] report the percentage share of the increase/decrease in choice probabilities; in other words, how much of the change in choice probabilities is attributed to either *Non-discounted* or *No purchase* individuals changing purchase behavior?

**Table 5:** Predicted change in choice probabilities given various price discounts.

	**Random utility maximization (RUM)**		**Random regret minimization (RRM)**		**Hybrid model (RUM + RRM)**	
	**Change in choice**	**Total**	**Change in choice**	**Total**	**Change in choice**	**Total**
	**probability (%)**	**change (%)**	**probability (%)**	**change (%)**	**probability (%)**	**change (%)**
**Panel (a)**						
*10% price discount*						
Discounted^a^	0.98	100.00	1.06	100.00	1.05	100.00
Non-discounted^a^	−0.55	−55.47	−0.63	−59.74	−0.62	−58.75
No purchase^a^	−0.44	−45.53	−0.43	−40.26	−0.43	−41.25
*50% price discount*						
Discounted^a^	5.01	100.00	5.31	100.00	5.28	100.00
Non-discounted^a^	−2.73	−54.39	−3.11	−58.53	−3.06	−57.89
No purchase^a^	−2.29	−45.61	−2.20	−41.47	−2.23	−42.11
**Panel (b)**						
*$3.75 price level*						
Price increase^a^	−6.24	−100.00	−6.79	−100.00	−6.67	−100.00
No–price increase^a^	3.55	56.82	4.20	61.87	4.04	60.51
No purchase^a^	2.69	43.18	2.59	38.13	2.64	39.49

a *Average effects, author calculations*.

Comparing changes in choice probabilities across behavioral models reveals several insights. First, all the signs on the coefficients are as hypothesized—a decrease in the price increases the choice probability in the *Discounted* store. Second, the changes in choice probabilities under the RRM framework are greater than RUM+RRM, which are greater than the RUM framework (e.g., 1.06 > 1.05 > 0.98 for a 10% decrease in price). As the price discount increases, this effect becomes more pronounced. Given either a 10 or 50% reduction in price, more individuals are predicted to purchase ground beef under the RRM framework than under the RUM framework. This aligns with our previous findings that choice attribute elasticities were more elastic under RRM compared to RUM (see Table 3). It also implies that understanding which behavioral framework applies to ground beef purchases could affect policy decision making and industry response. For example, knowing that choices are more sensitive to price changes under RRM than RUM, price would not need to be discounted as much to ensure the same market share. Thus, a 10% price discount under RUM would achieve the same change in choice probability as a 9.24% price discount under an RRM framework.

Third, a decrease in the price of the affected alternative incentivizes some individuals who were not purchasing ground beef to purchase ground beef. This is seen by a non-zero value for *No Purchase*: −0.43, −0.43, −0.44 under RRM, RUM+RRM, and RUM, respectively. For a 10% price discount 0.43, 0.43, and 0.44% of respondents would now purchase ground beef under the different respective frameworks. While the shift is slightly larger under an RUM framework, the share of the *No Purchase* change relative to the total change is ~45% (40%) of the increase in choice probability under the RUM (RRM) framework. This implies that under an RUM framework, more of the change in choice probability comes from people willing to switch from not purchasing ground beef to purchasing ground beef rather than shifting consumption between stores. Given that price is one product attribute in which stores compete, the results indicate that one store's prices have a larger negative/competing effect on another store under an RRM framework than under an RUM framework.

We now explore how stores engaging in testing after a food safety issue would affect consumer choice probabilities. Table 2 reported WTP estimates for *Private Testing* under both RUM and RRM frameworks. We take the average private testing premium between these frameworks, $1.50 per lb., and then add it to the average price of ground beef in our study and assume that price is fixed across shopping venues. We then set this as the price level and observe changes in choice probabilities. We interpret these changes in choice probabilities as measures of market share that a store could capture by engaging in additional store food safety testing. Table 5, panel (b), reports the changes in choice probabilities. At $3.75 per lb. ($2.25 + $1.50 testing premium), the choice probability would increase on average by 6%. Note that the sign is negative for the *Discounted store* since the price was raised.

## Discussion and Conclusions

This paper empirically examined how consumers perceive and value different classes of food safety recalls. Specifically, we compared *E. coli* (Class I) and antibiotic residue (Class II) ground beef recalls in the prior month. A hypothetical choice experiment was used where consumers selected among different shopping experiences and choice attributes that included type of recall, store format, and private testing. Data were modeled using two competing behavioral frameworks: the commonly assumed random utility maximization (RUM) framework and the newer random regret minimizing (RRM) framework.

Consumers were less likely to purchase ground beef given an *E. coli* recall in the prior month compared to an antibiotic residue recall. This was consistent with our hypothesis that consumer valuations for food safety recalls are a function of the relative probability of illness and death. The odds of purchasing ground beef were higher in supermarkets compared to club and convenience stores and if the store engages in private food safety product testing. Results were consistent across behavioral frameworks and model specifications.

We found that the RRM or the hybrid model (RUM + RRM) was the preferred model specification. The hybrid specification shows that about 60% of individuals are regret minimizer while only 30% maximize utility. If a RUM framework were used, this would imply 60% of individuals would be “misclassified.” The model significantly affected direct elasticity estimates, classification of consumers, and policy/industry price mechanisms. Results were consistent with previous studies that the behavioral assumption of random regret appears better suited to model risky choices having potential losses (for example, food poisoning). Likewise, results predicted behavioral responses may differ substantially conditional on behavioral framework.

While the magnitude of difference between behavioral frameworks is modest, the implied aggregate impact on ground beef consumption is economically significant. By comparing the relative discount of *E. coli* to antibiotic residue, an estimate of the necessary investment by the industry can be obtained. More importantly, this study speaks to policy and industry debates regarding the need to reduce antimicrobial use in livestock production, which could lead to reduced antibiotic residue in meat products and ultimately lower antimicrobial resistance in human health. Since consumers value antibiotic residues < *E. coli* contamination, any investment in research and development to reduce antibiotic residue in beef supply chain should be no more than 60% of the total research and expenditure of *E. coli*. However, given that antibiotic residue is generally known to not cause immediate health concerns, this estimate is likely an extreme upper bound. By how much likely depends on the relative weighting policy makers place on mitigating Class II recalls relative to Class I recalls given finite food safety funding. These results provide new, systematic evidence that both the class of food safety recall and the behavioral framework have substantial impacts on policy decisions and suggest that strengthening organizational trust has the potential to increase meaningfully the efficiency of research and development on antibiotic residue.

## Data Availability Statement

The raw data supporting the conclusions of this article will be made available by the authors, without undue reservation.

## Ethics Statement

This study involved human participants without deception and minimal risks. The study was approved by the IRB at Kansas State University. All participants were provided a written informed consent to prior to study participation.

## Author Contributions

ED generated the research question, designed the experiment, conducted the experiment, conducted the analysis, wrote, and edited the paper. KA designed the experiment, conducted the experiment, conducted the analysis, and edited the paper. JB designed the experiment, conducted the analysis, and edited the paper. TS designed the experiment, wrote and edited the paper.

## Conflict of Interest

The authors declare that the research was conducted in the absence of any commercial or financial relationships that could be construed as a potential conflict of interest.

## Footnotes

^1^For example, in the 1998 Colorado Boxed Beef *E. coli* recall, of 359,000 pounds of ground beef implicated in the recall, only one pound was ultimately recovered (1).

^2^Processed meat is subject to both random and targeted sampling for antimicrobial residue by FSIS's National Residue Program. While the number of samples violating residue levels remains low (~0.3% for livestock products) some products inevitably pass inspection, reach the supermarket and are later recalled due to consumer reports of adverse reactions or illnesses from product consumption.

^3^The frequent media links between Chipotle Mexican Grill and *E. coli* in 2015 caused sharp decrease in restaurant patronage reflected in stock price devaluation. The incubation period for *E. coli* is usually 3–4 days after the exposure, but may be as short as 1 day or as long as 10 days. Symptoms begin with a mild belly pain or non-bloody diarrhea which worsens over several days.

^4^People who self-identified as not eating meat completed the survey is significantly less times (18.5 min) than those who self-identified as meat eaters (25.8 min). This provides some evidence that individuals who did not eat meat products were less engaged in the survey, thus potentially downward biasing the results.

^5^“Other” stores include dollar stores, drug stores, natural + organic stores, convenience stores, ethnic food stores, and online-only food stores.

^6^Government included FSIS, FDA, CDC, and food industry scientists. Advocacy groups included consumer organizations and environmental groups. Producers included food manufactures, farmers/growers, and local butcher. Stores included my primary food store, fine dining restaurant, casual dining restaurant, and fast food restaurant. Media included TV, radio, newspapers, food magazines, food and cooking channels, social media, blogs, internet website, and entertainment industry. Family and Friends included friends, family, doctors, and health/dietary/life coach.

^7^The cheap talk stated the following “*Imagine you are thinking about going to a store to buy ground beef. Each store will have the following: a specific format (e.g., supermarket or club store) whether a food safety issue has ever occurred in the store (E. coli or antibiotic residue) whether additional private ground beef safety testing is conducted and the price for ground beef ($/lb). You will be shown two different stores and asked which one you would shop at, if any. Please answer as honestly as possible and in a manner that you think would reflect your true shopping attitude*.”

^8^We acknowledge that this potentially biases the results, as some consumers primarily purchase food at dollar, drug, organic/natural, convenience, ethnic, or online stores. However, ~95% of individuals who completed the survey indicated that supermarkets, club stores, and convenience stores were considered their primary source of grocery-type items. This is representative of the average U.S. consumer population.

^9^We recognize that not every store format may be able to conduct private testing. Reasons vary from logistic to cost-effectiveness. We do not attempt to answer if it is feasible for certain store formats to engage in testing and at what price premium this becomes cost-effective.

^10^After initial confirmation of a food safety outbreak, the CDC continues to monitor situation for about one month. CDC stops monitoring most food safety issues after one month since most of the harmful product has either been recalled or diminishing safety incidences. This does not imply that there are no more food safety issues but rather that they are not actively monitoring the situation anymore. Thus, 1 month is a broad way of defining the length of time to resolve food safety issues.

^11^Model estimations converged with set convergence criteria (1e-6) based on the gradient using starting values from the corresponding standard multinomial model in NLOGIT. We ran several robustness checks changing tolerance criteria algorithmic parameters, and found that both coefficient estimates and class size were robust to these changes.

^12^It could be possible that a specific consumer could be regret minimizing in some circumstances but maximize utility in others. Thus, our premise is not that consumers are either one or other but rather circumstances could arise, in our case a food recall in the previous month, where consumers wish to minimize regret for making choices that could lead to potentially negative consequences. This conclusion appears to align well with the increased sensitivity of U.S. population toward health and healthy foods in recent years.
