# Supplementary material for: Consumer Reactions to E. Coli and Antibiotic Residue Recalls: Utility Maximization vs. Regret Minimization
Source: Front Vet Sci. 2020 Sep 3;7:611. doi: 10.3389/fvets.2020.00611 (PMC7494847; doi:10.3389/fvets.2020.00611)
Supplement: Supplementary file 1 [file Table_1.DOCX]

**Consumer Reactions to E. coli and Antibiotic Residue Recalls: Utility Maximization vs. Regret Minimization**

**(Supplementary Material)**

**Appendix A – Survey Participant Demographic, Meat Consumption Habits, and Trust for Food Safety Information**

**Table A1. Demographics of Survey Participants**

|  | **Survey Participants (2017)**  **(N=1994)** | **Census Data (2011)** |
| --- | --- | --- |
| *Census Region* |  |  |
| Northeast | 17.7 | 17.5 |
| Midwest | 21.6 | 21.1 |
| South | 37.6 | 37.7 |
| West | 23.1 | 23.7 |
| *Biological Sex* |  |  |
| Female | 59.5 | 51.4 |
| *Age (yrs. old)* |  |  |
| 18-24 | 15.5 | 12.9 |
| 25-34 | 25.7 | 17.6 |
| 35-44 | 16.9 | 17.0 |
| 45-54 | 13.3 | 18.4 |
| 55-64 | 13.7 | 16.1 |
| 65-74 | 11.6 | 10.0 |
| 75 or older | 3.2 | 8.0 |
| *Household Information* |  |  |
| Married | 51.5 | n/a |
| Children under 12 in Household | 32.1 | 33.4 |
| SNAP (food stamp) participant | 17.5 | 18.4 |
| College Degree | 38.7 | 29.3 |
| *Income (USD)* |  |  |
| less than $20K | 15.6 | 11.7 |
| $20K-$39K | 23.5 | 17.6 |
| $40K-$59K | 19.8 | 15.7 |
| $60K-$79K | 14.3 | 13.5 |
| $80K-$99K | 10.9 | 10.3 |
| $100K-$119K | 5.5 | 8.1 |
| $120K-$139K | 3.2 | 6.1 |
| $140K-$159K | 2.8 | 4.3 |
| $160K or higher | 4.4 | 12.7 |
| *Race* |  |  |
| Hispanic | 7.9 | 16.9 |
| White | 75.7 | 73.8 |
| Black | 8.9 | 12.6 |
| Asian | 5.8 |  |
| Other | 1.8 |  |

Notes: ^a^ Percent of total responses within a given information medium; ^b^ Other includes dollar, drug, natural, organic, convenience, ethnic, or online food stores.

**Table A2. Meat Consumption Habits**

| *Meat Product* | **Never** | | **Once a month or less** | | **2-3 times per month** | | **Once per week** | | **More than once per week** | |
| --- | --- | --- | --- | --- | --- | --- | --- | --- | --- | --- |
|  | *N^a^* | *(%)^b^* | *N* | *(%)* | *N* | *(%)* | *N* | *(%)* | *N* | *(%)* |
| Beef^c^ | 32 | (1.60) | 227 | (11.38) | 338 | (16.95) | 493 | (24.72) | 904 | (45.34) |
| Pork | 107 | (5.37) | 465 | (23.32) | 480 | (24.07) | 541 | (27.13) | 401 | (20.11) |
| Chicken | 14 | (0.70) | 99 | (4.96) | 254 | (12.74) | 482 | (24.17) | 1145 | (57.42) |
| Turkey | 73 | (3.66) | 1024 | (51.35) | 345 | (17.30) | 280 | (14.04) | 272 | (13.64) |
| Fish | 245 | (12.29) | 632 | (31.70) | 432 | (21.66) | 394 | (19.76) | 291 | (14.59) |
| Other | 830 | (41.62) | 510 | (25.58) | 227 | (11.38) | 209 | (10.48) | 218 | (10.93) |

Notes: ^a^ Number of individuals; ^b^ Percentage of the 1994 individuals in the survey (N=1994); ^c^ Rows sum to 1994 (100%).

**Table A3. Shopping and Cooking Habits by Individuals Primary Store for Food Purchases**

|  | **Cooking Habits (%)^a^** | | | |
| --- | --- | --- | --- | --- |
|  | *I cook* | *We cook* | *They/Someone else cooks* | *Total* |
| **Supermarket** |  |  |  |  |
| *Shopping Habits* |  |  |  |  |
| All | 32.64 | 4.36 | 1.20 | **38.2**^d^ |
| Majority | 6.57 | 8.53 | 1.96 | **17.06** |
| Equal | 0.90 | 3.26 | 1.40 | **5.56** |
| Minority | 0.50 | 2.26 | 1.81 | **4.57** |
|  | **40.61**^c^ | **18.41** | **6.37** | **65.39**^e^ |
| **Club** |  |  |  |  |
| *Shopping Habits* |  |  |  |  |
| All | 14.54 | 2.55 | 0.60 | **17.69** |
| Majority | 3.01 | 4.36 | 0.50 | **7.87** |
| Equal | 0.55 | 1.2 | 0.65 | **2.4** |
| Minority | 0.05 | 0.5 | 1.35 | **1.9** |
|  | **18.15** | **8.61** | **3.1** | **29.86** |
| **Other**^b^ |  |  |  |  |
| *Shopping Habits* |  |  |  |  |
| All | 2.15 | 0.35 | 0.25 | **2.75** |
| Majority | 0.30 | 0.65 | 0.25 | **1.2** |
| Equal | 0.05 | 0.3 | 0.05 | **0.4** |
| Minority | - | 0.2 | 0.15 | **0.35** |
|  | **2.5** | **1.5** | **0.7** | **4.7** |

Notes: ^a^ Reported as percent of individuals (N=1994). Entire table sums to 100; ^b^ Other includes dollar stores, drug stores, natural + organic stores, convenience stores, ethnic food stores, and online-only food stores; ^c^ Sum of a specific cooking habit across all shopping habits but within a given store format; ^d^ Sum of a shopping habit across all cooking habits but within a given store format; ^e^ Sum across all shopping and cooking habits but within a given store format

**Table A4. Individuals Trust for Food Safety Information**

| *Helpfulness for Food Safety Information*^a^ | **Helpful** | | **Somewhat Helpful** | | **Not helpful** | |
| --- | --- | --- | --- | --- | --- | --- |
|  | *N*^b^ | *(%)*^c^ | *N* | *(%)* | *N* | *(%)* |
| Government^d^ | 548 | (27.5) | 616 | (30.9) | 830 | (41.6) |
| Advocacy Groups | 0 | (0.0) | 261 | (13.1) | 1733 | (86.9) |
| Producer | 222 | (11.1) | 540 | (27.1) | 1232 | (61.8) |
| Store | 88 | (4.4) | 382 | (19.2) | 1524 | (76.4) |
| Media | 112 | (5.6) | 669 | (33.5) | 1213 | (60.9) |
| Family and Friends | 374 | (18.8) | 773 | (38.7) | 847 | (42.5) |

Notes: ^a^ *Government* included SDA-FSIS, FDA, CDC, and food industry scientists. *Advocacy Groups* included consumer organizations and environmental groups. *Producers* included food manufactures, farmers/growers, and local butcher. *Stores* included my primary food store, fine dining restaurant, causal dining restaurant, and fast food restaurant. *Media* included TV, radio, newspapers, food magazines, food and cooking channels, social media, blogs, internet website, and entertainment industry. *Family* *and Friends* included friends, family, doctors, and health/dietary/life coach; ^b^ Number of individuals; ^c^ Percentage of the 1994 individuals in the survey; ^d^ Rows sum to 1994 (100%).
